# A Spectral Principal Component Analysis-Based Framework for Composite Hard/Soft Tissue Fluorescence Image Investigation

**DOI:** 10.3389/fphys.2022.899626

**Published:** 2022-07-13

**Authors:** Marie Piriou, Corinne Lorenzo, Isabelle Raymond-Letron, Sophie Coronas-Dupuis, Laetitia Pieruccioni, Jacques Rouquette, Christophe Guissard, Jade Chaumont, Louis Casteilla, Valérie Planat-Benard, Philippe Kemoun, Paul Monsarrat

**Affiliations:** ^1^ Dental Faculty and Hospital of Toulouse—Toulouse Institute of Oral Medicine and Science, CHU de Toulouse, Toulouse, France; ^2^ Restore Research Center, Université de Toulouse, INSERM 1301, CNRS 5070, EFS, ENVT, Toulouse, France; ^3^ LabHPEC, Université de Toulouse, ENVT (Ecole Nationale Vétérinaire de Toulouse), Toulouse, France; ^4^ Artificial and Natural Intelligence Toulouse Institute ANITI, Toulouse, France

**Keywords:** fluorescence, spectral microscopy, mineralized tissues, periodontal structures, algorithmic processing, principal component analysis

## Abstract

Traditional thin sectioning microscopy of large bone and dental tissue samples using demineralization may disrupt structure morphologies and even damage soft tissues, thus compromising the histopathological investigation. Here, we developed a synergistic and original framework on thick sections based on wide-field multi-fluorescence imaging and spectral Principal Component Analysis (sPCA) as an alternative, fast, versatile, and reliable solution, suitable for highly mineralized tissue structure sustain and visualization. Periodontal 2-mm thick sections were stained with a solution containing five fluorescent dyes chosen for their ability to discriminate close tissues, and acquisitions were performed with a multi-zoom macroscope for blue, green, red, and NIR (near-infrared) emissions. Eigen-images derived from both standard scaler (Std) and Contrast Limited Adaptive Histogram Equalization (Clahe) pre-preprocessing significantly enhanced tissue contrasts, highly suitable for histopathological investigation with an *in-depth* detail for sub-tissue structure discrimination. Using this method, it is possible to preserve and delineate accurately the different anatomical/morphological features of the periodontium, a complex tooth-supporting multi-tissue. Indeed, we achieve characterization of gingiva, alveolar bone, cementum, and periodontal ligament tissues. The ease and adaptability of this approach make it an effective method for providing high-contrast features that are not usually available in standard staining histology. Beyond periodontal investigations, this first proof of concept of an sPCA solution for optical microscopy of complex structures, especially including mineralized tissues opens new perspectives to deal with other chronic diseases involving complex tissue and organ defects. Overall, such an imaging framework appears to be a novel and convenient strategy for optical microscopy investigation.

## 1 Introduction

Optical microscopy is a *sine qua non* tool to investigate a wide range of physiological/pathophysiological processes within integrated biological systems. Required imaging scales and techniques depend on sample size, the ultimate resolution needed, i.e., the smallest feature to be distinguished, and the image contrast to be obtained. Traditional serial thin sectioning, delivering section thickness of a few microns, is the most popular technique. It not only provides very high optical resolution using conventional or confocal microscopy but also the possibility of performing histochemistry and immunostaining imaging with a plethora of specific antibodies.

The gold standard for mineralized large-sample microscopic investigation remains the use of histochemistry staining (e.g., hematoxylin-eosin, Masson’s trichrome, Goldner), requiring tissue decalcification, mostly with acids (e.g., nitric, hydrochloric, ethylenediaminetetraacetic acids) for several weeks (Valentine and Piper, 2012). In some cases, these treatments may complicate the histopathological analysis, especially for highly-mineralized bone or dental tissues that partially or completely disappear from the microscopic sections. In addition, the demineralization step may alter tissue morphologies and even damage the most fragile soft tissues [especially epithelia or structures undergoing tissue repair (Donath and Breuner, 1982; Callis and Sterchi, 1998)]. Moreover, the assessment of non-mineralized bone matrix deposits (for bone remodeling investigation during pathological processes or tissue regeneration, for example) is compromised after acid use involving lack of mineralized structure delineation (Baron, 1983; Bourque et al., 1993). Thus, several bright-field microscopy protocols that do not require decalcification have been proposed. After tissue fixation, large specimens are embedded in epoxy resin then cut with a diamond wire saw to be worn down for histochemical staining. However, the process of polymerizing methyl methacrylate for epoxy embedding requires high-heat application, thereby compromising the shape of cells and soft tissues, and are generally designed for specific items such as evaluation of biomaterials osseointegration (Park et al., 2015).

**FIGURE 1:**
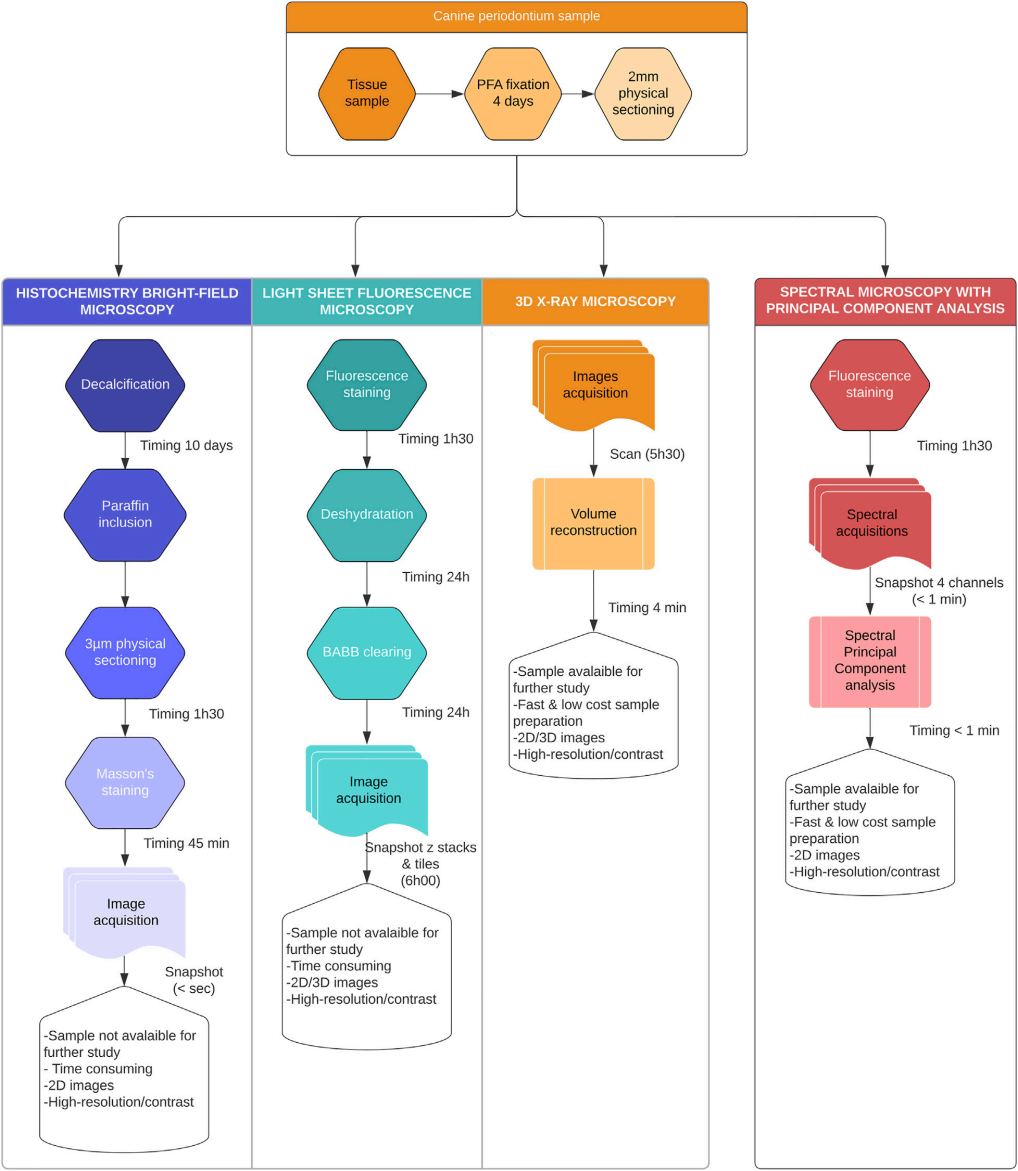
Summary of the sample processing. All samples were processed using conventional bright-field microscopy, 3D X-Ray microscopy, Light Sheet microscopy (LSM), and compared to the Spectral Principal Component Analysis (sPCA). Pros and cons were presented for each method, revealing the unique contribution of sPCA as a fast and scalable technique.

Considering the technical challenges required for traditional serial thin sectioning approaches, especially for composite soft/hard tissues, alternative workflows avoiding sample decalcification have been developed (Figure 1). Recently, various fluorescence microscopy techniques, including confocal scanning or light sheet fluorescence microscopy (LSFM), combined or not with clearing methods, have been proposed for composite structures (including dental and bone tissue) imaging (Kabasawa et al., 1995; Ye et al., 2016; Hong et al., 2019). Confocal microscopy, useful for high-resolution analysis, generates 3D images for about a few hundred microns (100–200 μm) of sample depth. Nevertheless, it is not suitable to recover structural information for an entire organ system, such as the whole tooth. On the other hand, LSFM enables large-sample volumetric imaging (Hong et al., 2019) but typically requires clearing (Jing et al., 2019), leading to tissue distortion, fluorescence quenching and 3D structural information loss. In addition, large-scale cleared samples imaging can easily produce gigabytes or even terabytes of data, a great challenge for both software and hardware. Alternatively, X-ray micro-tomography (nano or micro-CT) acquisitions are commonly used to investigate hard tissues (du Plessis et al., 2017). However, soft tissue assessment by micro-CT requires contrast enhancement using heavy-metal stains (Pauwels et al., 2013). Moreover, the acquisition time of this method exceeds hours or days, especially for high-contrast imaging (du Plessis et al., 2017).

To overcome the limitations of the above-mentioned approaches for composite hard/soft tissue imaging, our aim was to develop fast, versatile, and reliable method, adapted for highly mineralized tissue visualization and providing a large field of view (>15 mm), good spatial resolution (∼5–10 µm), and contrast suitable for histological interpretation with a high level of detail. To meet this challenge, we proposed a synergistic and original approach based on multiple staining, wide-field fluorescence imaging, and spectral Principal Component Analysis (sPCA). We demonstrated the ability of our method to enhance feature extraction for large samples with composite soft/mineralized tissues, with particular emphasis on the periodontium or tooth-supporting tissue, a complex mineralized and non-mineralized composite structure.

## 2 Materials and Methods

### 2.1 Tissue Sample Preparation

Toothed hemi-mandible block-sections from two 13-year-old female Beagles (deceased from natural causes, without systemic pathology) were recovered from the LabHPEC (Histopathological Laboratory of the National Veterinary School of Toulouse). Anatomical pieces were immediately immersed in 10% neutral buffered formalin fixative after sampling for 4 days before storage in PBS at 4°C. Enamel was removed using dental burs and two- mm thick sections were obtained using a diamond wire saw under irrigation following the main axis of the premolar and/or molar roots. Thick sections were distributed alternatively between the non-decalcified experimental protocol required for spectral analyses and the standard protocol with decalcification to perform control histopathological histochemistry evaluations (Figure 1).

### 2.2 Multiple Staining of 2 mm Thick Sections

Sulforhodamine B (SRB, ref: S1402-16) was purchased from Sigma Aldrich (Burlington, MA, United States). Calcein-AM (ref: 65-0853-78), TO-PRO™-3 Iodide (TO-PRO3, ref: T3605), Wheat Germ Agglutinin-Texas Red™-X conjugate (i.e., WGA, ref: W21405) and 5 (6)-CFDA, SE; CFSE [5-(and-6)-Carboxyfluorescein Diacetate, Succinimidyl Ester], mixed isomers (CFDA-SE, ref: C1157) were purchased from ThermoFisher Scientific (Waltham, MA, United States). Non-decalcified thick sections were incubated with a mixture of dyes including 2 μg/ml of SRB, 50 µM of Calcein, 2 µM of TO-PRO3, 2 μg/ml WGA, 200 µM CFDA-SE for 1 h at room temperature. All details were presented in Supplementary Table S1. After washing thoroughly in phosphate-buffered saline (PBS) solution, the sections were imaged by fluorescence microscopy (Figure 1).

### 2.3 Fluorescence Image Acquisitions

**FIGURE 2:**
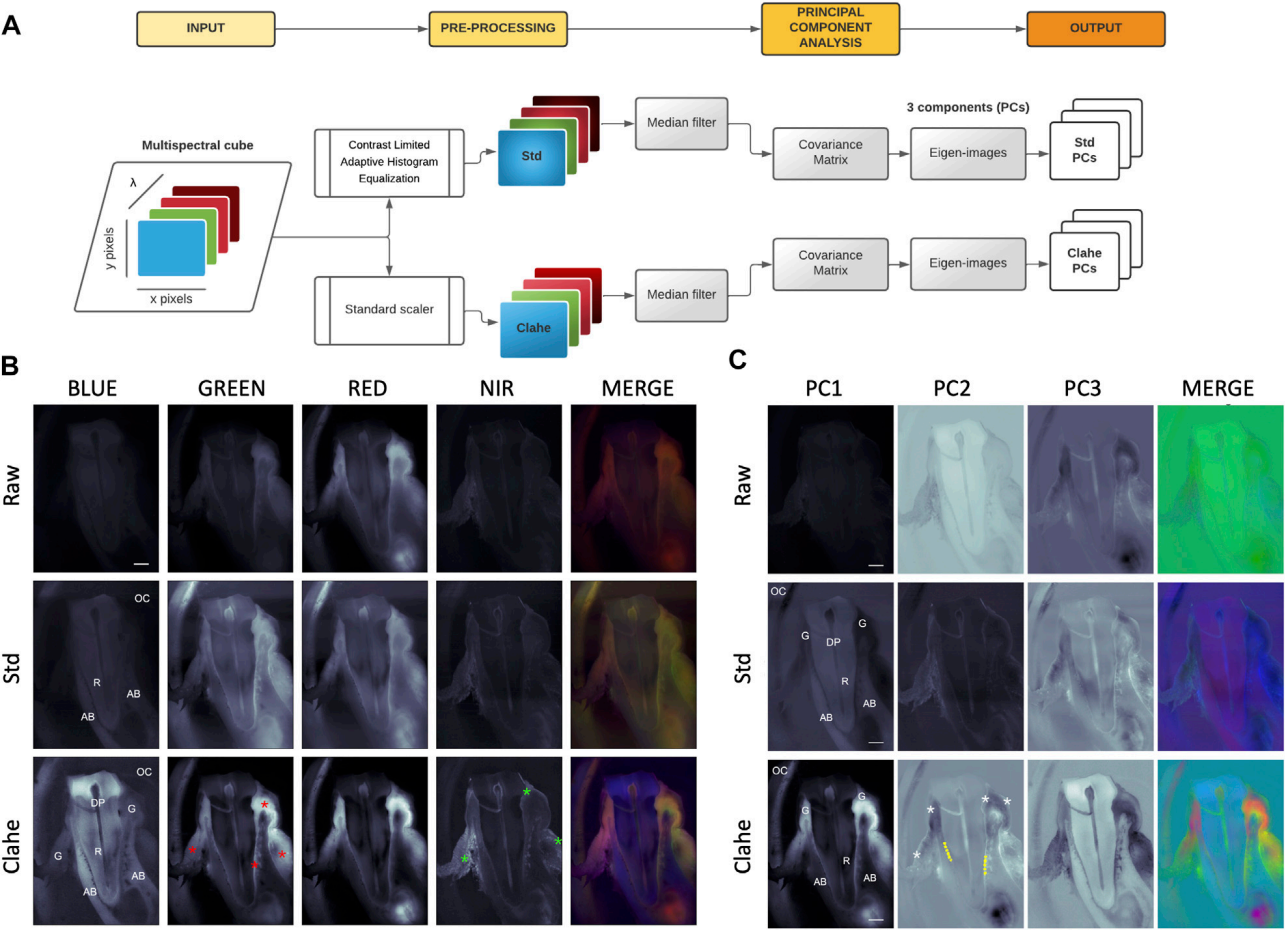
Spectral Principal Component Analysis (sPCA) workflow. **(A)**—sPCA workflow involves preprocessing of fluorescence acquisitions with standard scaler (Std) or Contrast Limited Adaptive Histogram Equalization (Clahe) including a median filter, followed by a Principal Component Analysis to derive 3 eigen-images. **(B)**—Std and Clahe preprocessing optimize the visualization of canine tooth root (R) and alveolar bone (AB) on a UV autofluorescence channel, and soft tissues on the other channels (red stars). Cell nuclei were particularly revealed by TO-PRO3 labeling in the NIR channel (green stars). OC: Oral Cavity. G: gingiva. DP: dental pulp. **(C)**—Top three eigen images derived from principal component analysis. Altogether, eigen-images from Std and Clahe greatly optimized hard [tooth root (R) and alveolar bone (AB)] and soft tissue discrimination [epithelia (*), gingival stroma G, periodontal ligament (PDL, dashed lines) and dental pulp (DP)]. OC: Oral cavity. Scale bar: 2000 µm.

A multi-zoom macroscope MacroFluo Z16 APO (Leica, Germany) was equipped with 4 filter blocks for excitation under UV, blue, green, and red light, and a respective collection of blue, green, red, and NIR emission light (see Supplementary Table S2 for wavelengths; Figure 2A). The exposure times were fixed at UV: 1,500 ms, Blue: 1,000 ms, Green: 500 ms, and Red: 2000 ms. The total magnification was obtained by combining the lens Plan Fluor 0.5x and setting the optical zoom. Images were acquired with a CoolSNAP ES cooled CCD camera (Roper Scientific Photometrics Instruments) with 1392*1040 pixels and a pixel size of 6.45*6.45 µm. Metaview software [Universal Imaging Corporation, Molecular Devices] was used for the control of the CCD camera. For simplification, we named the different acquisition channels by their emission light (blue, green, red, and NIR).

### 2.4 Image Processing and Analysis

Preprocessing: Two different methods were used (Figure 2A): a standard scaler (Std) and a Contrast Limited Adaptive Histogram Equalization (Clahe). The Std method was used to standardize each acquisition by subtracting the mean value of the pixels along the *x*-axis, divided by the standard deviation along this same *x*-axis (*scale* function from *scikit-learn 0.24.2*). Clahe, a local contrast modification algorithm, was applied from the *OpenCV* library [with a clipLimit of 12.0 and a tileGridSize of (10, 10)]. In both instances, a median filter of size 4 was passed afterwards, to reduce noise (*scipy 1.6.3*).

Principal Component Analysis (PCA): To define a spectral signature, a principal component analysis was performed on the spectral cube (*scikit-learn*). Each channel acquisition was considered as an 1D vector by concatenating image pixels in row-major order, achieving a dimensionality equal to the pixel count of the images (1392*1040 pixels, i.e., a dimensionality of 1,447,680 for the vector space). Three principal components were retained. The derived eigen-vectors were then rearranged back from the 1D vector to reconstruct 3 rectangular eigen-images. Finally, the values were normalized between 0 and 255 and the principal components 1, 2 and 3 considered respectively as the red, green, and blue channels in the RGB system to obtain a merged color image (Figure 2A).

The python code, including the preprocessing and PCA steps, is available on GitHub (https://github.com/paulmonsarrat/spectral_periodontal_tissues).

### 2.5 Histology on Decalcified Tissues Samples

To confirm the identification of tissue structures by spectral analysis, control sections from the same sites were routinely processed. Briefly, all formalin fixed 2-mm sections samples adjacent to fluorescent ones were decalcified with a commercial solution (Decalc®-Histolab) containing hydrochloric acid at 10%–20% for 10 days and rinsed before paraffin inclusion. After paraffin embedding, 3 µm thickness paraffin sections were dewaxed (successive toluene and descending alcohols bathes) and stained with Haematoxilin and Eosin and Masson trichrome. The stained slides were imaged by light microscopy on a Nikon Eclipse Ci-L microscope with a DS-Fi3 Camera and NIS Elements D software.

### 2.6 Micro-CT Imaging

Micro-CT acquisitions were performed using the Zeiss Versa 510 laboratory X-ray microscope (Carl Zeiss Meditec, France) at 40 kV and 3 W acquisition settings for an isotropic voxel size of 18.7 µm (0.4× lens) with a LE1 tube output filter. A targeted acquisition at higher magnification on the alveolar bone crest was also performed (40 kV, 3 W, 1.5 µm voxel size, and LE1 output filter). 1601 projections were acquired, for a total of 3.3 GB raw data for each acquisition, and volumes automatically reconstructed then visualized using ImageJ^®^ 1.53 software.

### 2.7 Light Sheet Fluorescence Microscopy Imaging

PFA-fixed 2 mm sections were embedded in 1% Low Melting Agarose before clearing. Sections were dehydrated with 100% Methanol (MeOH) baths (4, 12, and 8 h). The clearing process was initiated with a 1 vol. Benzyl Alcohol (BA)/2 vol. Benzyl Benzoate (BB)/3vol. MeOH for 30 min. Finally, the sections were placed in a bath of 1 vol. BA/2vol. BB bath, renewed twice every 12 h. All the steps of the clearing process were performed at room temperature, under agitation, and in the dark.

The cleared samples were imaged in a quartz cell containing BABB solution. The latter was placed in a custom LSFM based on cylindrical lens illumination (0.6×) with wavelengths of 405 nm, 488 nm, 642 nm (Stradus), and 561 nm (Coherent), and horizontal macroscopic detection as described in (Kennel et al., 2020).

The light sheet thickness (T) was set to 35 μm to accommodate the largest field of view (FOV) at the lowest magnification (i.e., 1.2×). The macroscope was a Nikon AZ100M macroscope with a 2× magnification air lens, providing a zoom factor of 8. The final magnification of 1.8× used here gives an estimated lateral resolution of 3.6 μm against an axial extent of the light sheet of approximately 5 μm. Detection was performed with bandpass filters: 447/60 (405 nm laser) and 609/57 (561 nm laser) or longPass: 488/LP (488 nm laser) and 647/LP (642 nm laser) mounted in a filter wheel (LB10, Sutter Instruments, Novato, CA, United States) and an sCMOS camera (ORCA Flash4.0, Hamamatsu, Japan). The whole tissue acquisition consisted of a 1500*2048 xy z-image stack, with a voxel resolution of 3.38 µm/pixel for xy and 5 µm/pixel for z, for a total of 282 GB raw data.

## 3 Results

### 3.1 Sample Staining and Image Acquisitions

The starting point for fluorescent staining optimization was to set up a labeling agent combination to maximize subtle discrimination of the largest possible number of periodontal structures. Different dye mixes, with various wavelengths, either as free or as conjugated dyes, were assessed for their ability to discriminate close tissues such as dentin, cementum, periodontal ligament (PDL) and alveolar bone, and at the same time clearly highlight gingival tissue (Supplementary Table S2).

The dyes were selected by their putative chemical affinity and their resultant capability to highlight multi-tissular contrast at a suitable signal-to-noise ratio. Dye concentration, time, and incubation temperature were optimized to obtain an adequate inter-tissular contrast on the 2-mm thick sections. Fluorescence multispectral datasets (Figure 2B) were acquired using sequential imaging with several excitation wavelengths, combined with optical filtering of the fluorescence signal by ease rotating a filter wheel in front of a CCD camera. Natural autofluorescence contribution, requiring high exposure time, was significantly lower than fluorescence labelling in all the channels, and did not sustain appropriate discrimination between tissular componants (Supplementary Figures S1). Single-labeling showed fluorescence spectral overlap between the different dyes used (Supplementary Figures S2, S3). Supplementary Figure S2 illustrates the differential tissue staining between Calcein-AM and CFDA-SE dyes with a clearer contrast of mineralized tissue compared to soft tissue, particularly brought out by CFDA-SE. Both dyes exhibit ester terminations whose role is usually to be cleaved by the cellular esterases of living cells while emitting a green fluorescence signal. Although the maximum fluorescence emission of Calcein-AM and CFDA-SE requires the esterase activity of living cells, the presence of residual and non-specific esterase activity even after tissue fixation has been shown (Cannon et al., 1982). SRB are small and highly polar molecules that may diffuse passively across connective tissues (Appaix et al., 2012). SRB spectral contribution highlighted a tooth and bone signal on the green channel and connective tissues on the red channel (Supplementary Figure S3). WGA Texas Red™-X conjugate has a high binding affinity for proteoglycans, primarily binding to N-acetylglucosamine residues (Goldstein et al., 1997). Although WGA Texas Red™-X conjugate’s single contribution provided a weak red channel signal, the contrast between the green and red channel highlighted mineralized structures (Supplementary Figure S3). TO-PRO3 dye is a carbocyanine monomeric nucleic acid with far red fluorescence. It is usually used as a nucleic counter stain and dead cell indicator considered one of the most sensitive probes for nucleic acid detection, including the paraffin section of soft tissues (Bink et al., 2001). TO-PRO3 provided complementary contributions, including nuclei distribution across the different channels (Supplementary Figure S3). Finally, multi-staining revealed that the contribution of each dye (Calcein-AM, CFDA-SE, WGA, SRB, and TO-PRO3), gathered in a mix, and pre-processed in an optimal way by both Std and Clahe, provides significant information for canine periodontium microscopic investigation (Figure 2B).

### 3.2 Fluorescence-Based Acquisitions Spectral Analysis

The main objective of this computational analysis was to highlight, for each channel, the contrast distinction among the periodontal tissues to define a fluorescence fingerprint rather than to carry out signal intensity differential canonic analysis among the different channels. An overview of the algorithm pipeline is shown in Figure 2A. First, the raw images obtained in the different channels undergo a preprocessing step required to resample the signal and allow a differential analysis of tissues while maintaining a maximum signal- to- noise ratio. Figure 2B illustrates the complementarity between both preprocessing strategies used: the Std scaler and the Clahe. The Std scaler resamples the images line by line and better highlights the contrasts within the different tissues encountered on the same line. This explains the most important noise in the image portions where there is no structure of interest (Figure 2B, Std row). On the other hand, Clahe (Figure 2B, Clahe row) allows an adaptive histogram equalization of the image tile by tile, followed by a bilinear interpolation that removes artifacts in tile borders. This transformation makes it possible to highlight the inter-structure contrast for each channel with a higher signal-to-noise ratio than Std. From the Std scaler and Clahe images generated, a dimensionality reduction by PCA was used to derive 3 “eigen-images,” considered the unique tissue spectral signature. Figure 2C clearly exhibits the benefit of preprocessing and the complementarity of the Std and Clahe approaches that emphasize distinct anatomical elements. The algorithmic process definitely optimized the tissue structure identification compared to the fluorescence microscopy alone (Figure 2B). As a result, PCA dimensionality reduction highlighted all the tissue structures with a particular focus on hard tissues and epithelium from the Std scaler, while stroma was mainly enhanced by Clahe.

### 3.3 Highlighting Soft/Hard Tissue Structures and Morphologies

**FIGURE 3:**
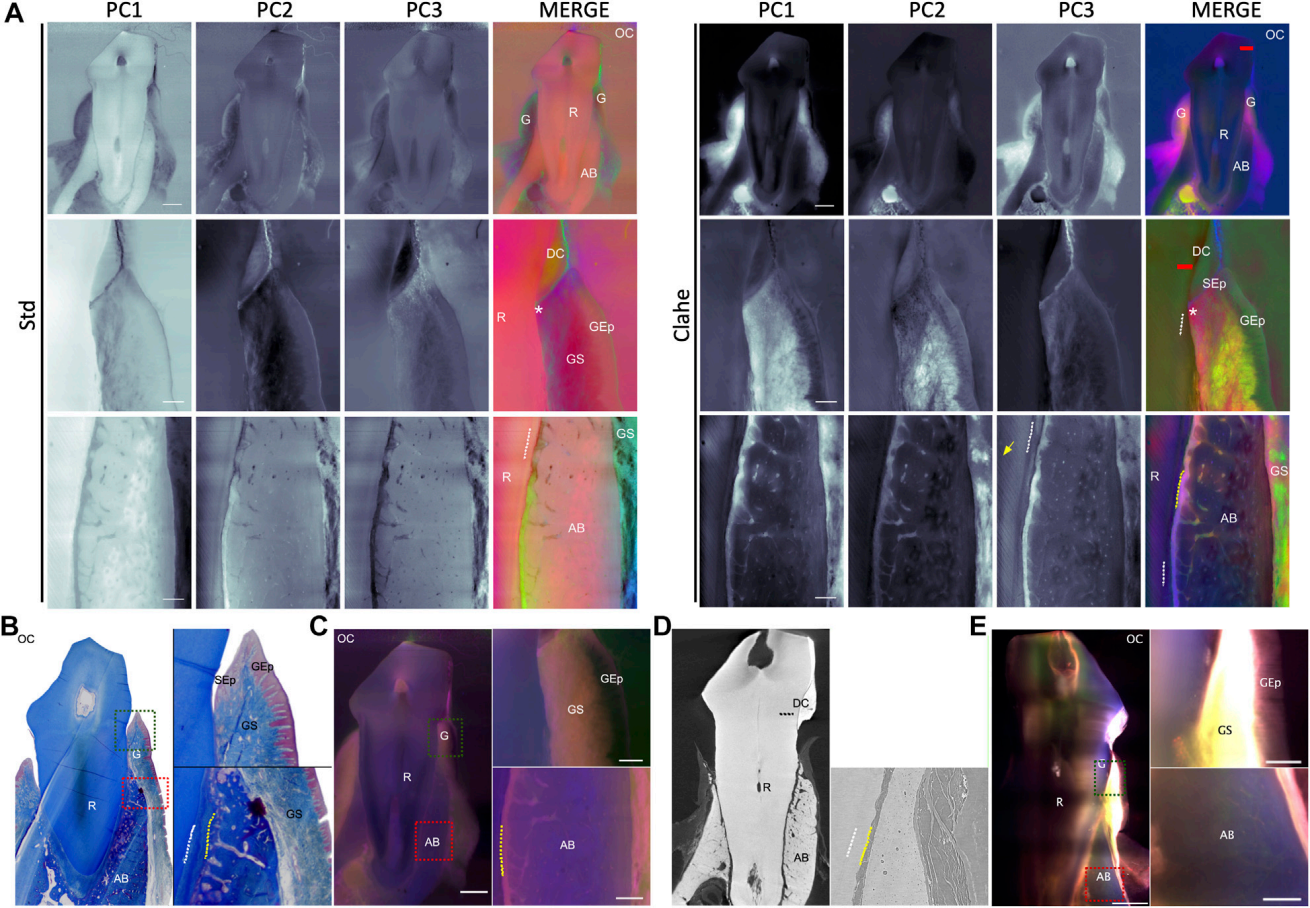
Canine periodontal tissue identification by spectral Principal Component Analysis is confirmed by conventional microscopy approaches. **(A)**—2-mm thick fluorescence imaging of canine periodontal sample and spectral Principal Component Analysis (sPCA) after Std or Clahe preprocessing. Interestingly, sPCA enhances root cementum (white dashed lines), gingival stroma (GS) and periodontal ligament (PDL, yellow dashed lines), alveolar bone (AB), keratin on epithelium surface (green, Std, merge), intra-dentinal tubuli (orange arrow) and dental calculus (DC). OC: oral cavity. R: tooth root. G: gingiva. Gep: gingival epithelium. Sep: Sulcular epithelium. Red line: enamel-cementum junction. *: dento-gingival junction. Scale bar: 2000 µm (low magnification), 500 µm (high magnification). **(B)**—Masson’s trichrome staining of decalcified 3 µm thickness sections at low (×10) and higher (×100) magnification by optical microscopy. **(C)**—2-mm-thick periodontal sample fluorescence multi-staining imaging with optical macroscope (LEICA^®^ MacroFluo Z16 APO). Scale bar: 2000 µm. **(D)**—2-mm thick 3D X-ray imaging of canine periodontal sample using Zeiss Versa 510 microscope at low (×0.4) and higher (×4) magnification. **(E)**—2-mm thick imaging of canine periodontal sample by Light Sheet Fluorescence Microscopy after BABB clearing.

At low magnification, mineralized [basal, alveolar bone (AB), tooth root, calculi] and soft (PDL and gingival) tissues, gingival sulcus (groove between the gingiva and the initial part of the tooth root), as well as anatomical landmarks (dentogingival junction, cementoenamel junction or root apex) are clearly highlighted by the eigen-images derived from Std and Clahe preprocessing (Figure 3A). Spectral decomposition by PCA was useful to point out the basal layers and acanthosis. The dentogingival sulcus was easily delineated. In this area, the sulcular epithelium, as well as the junctional epithelium with its adhesion interface to the root cementum, can also be identified. Among the different eigen-images of the PCA decomposition, there is always one allowing optimal discrimination of periodontal connective tissues with gingival and PDL matrix fibers distribution, suitable to evaluate the stroma homogeneity or possible fibrosis. Interestingly, this contrast enhancement process allows for rapid and accurate evaluation of dental and periodontal hard tissue morphology and arrangement, including osteoplastic lacunae or root cementum, critical to assess periodontal health or regenerative therapy outcomes. The PDL interface joint between the cementum and alveolar bone was clearly pointed out (Figure 3A, yellow dashed line). In some cases, at high magnification, even the dentin tubules were located. Conventional microscopy approaches consistently confirmed these tissue identifications, localizations and morphologies (Figures 3B–E). Indeed, gingival epithelial and stroma tissue organization was mainly highlighted by Masson’s trichrome on decalcified samples (Figure 3B), while root-cementum anchorage morphology was likely pointed out by 3D X-rays (Figure 3D) and LSFM (Figure 3E) acquisitions. Without contrast enhancement, non-decalcified fluorescence multi-staining samples imaged with the optical microscope did not display satisfactory hard and soft tissue discrimination (Figure 3C).

### 3.4 Application of Periodontal Microscopy in Large Animal Samples

#### 3.4.1 Spectral Analysis of the Whole Canine Periodontium

**FIGURE 4:**
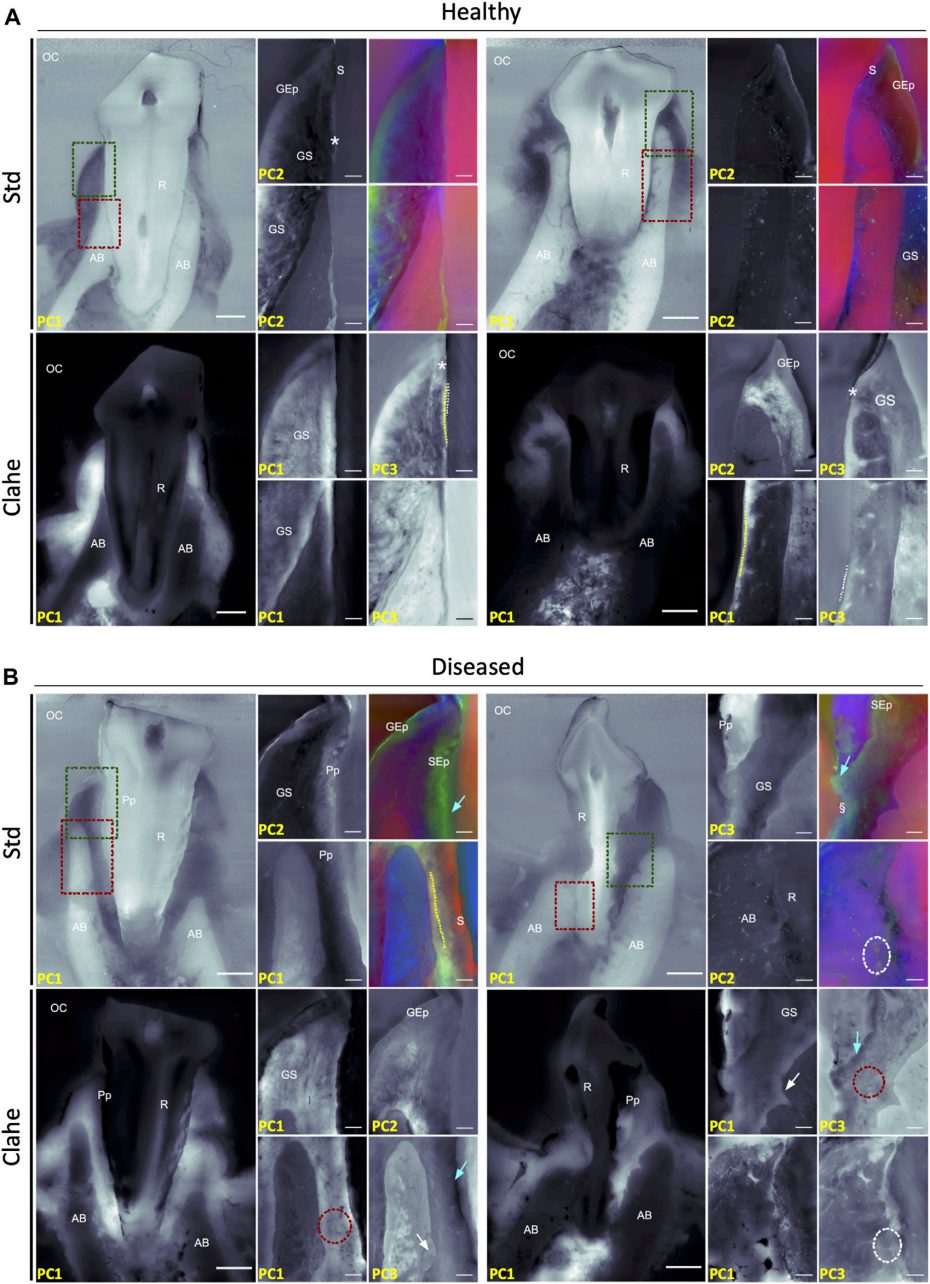
Spectral analysis of the healthy and diseased canine periodontium. For each case, the eigen-image the most contributive for histopathological analysis was presented. **(A)**—Short gingival sulcus (S), regular acanthosis in the marginal gingival epithelium (GEp), adherent junctional epithelium (*), homogenous gingival stroma (GS), regular-thickness cement (white dashed lines) and PDL (periodontal ligament, yellow dashed lines), and non-resorbed alveolar bone (AB) in canine healthy periodontium. **(B)**—Periodontal pocket (Pp) with deep gingival sulcus exhibiting non-regular acanthosis involving sulcular epithelium (SEp), long junctional epithelium (blue arrows), heterogeneous fiber arrangement in gingival stroma, enlarged PDL thickness (red dashed circles) with resorbed (white arrows) and/or ankylosed (white dashed circles) root and bone in diseased periodontium. Note the interface between the long junctional epithelium and the root at the bottom of the periodontal pocket (§). OC: oral cavity. R: tooth root. Scale bar: 2000 µm (low magnification), 500 µm (high magnification).

Given the validation of tissue investigation provided by the contrast enhancement as described above, canine periodontium health may be assessed at the microscopic level using the proposed spectral analysis framework. A dento-gingival junction localization close to the cementoenamel junction (CEJ), a short gingival sulcus, cementum-to-bone joint with a non-resorbed AB and a regular PDL thickness on the periphery of the tooth root, typical of a healthy periodontium, was easily highlighted (Figure 4A). Given the advanced age of the canine subjects under study, it was not surprising that the AB level was mainly located at the middle of the root instead of near the CEJ, as in young subjects. Diseased periodontium was plainly pointed out by highlighting typical periodontal pockets with a deep gingival sulcus and enlarged PDL thickness with resorbed and/or ankylosed root and bone (Figure 4A).

#### 3.4.2 Spectral Analysis of the Superficial Periodontium (Gingiva)

Spectral analysis confirmed steady linearity and thickness of the gingival epithelia in healthy periodontium, with regular acanthosis confined to the gingival margin epithelium. The gingival sulcular epithelium, as well as the junctional epithelium with its adhesion interface to the root cementum, can be identified (Figure 4A). In diseased periodontium (Figure 4B), processed images highlighted the absence of the junctional epithelium adhesion on the root cementum and, therefore, the presence of a periodontal pocket with non-regular acanthosis that newly occurred in the sulcular epithelium. Interestingly, the investigation of the interface between the long junctional epithelium at the bottom of the pocket, typical of a diseased periodontium, and the cementum surface through the contrast provided between the epithelial soft and hard connective tissues, was particularly convenient (Figure 4B). Whereas the gingival stroma and fibers arrangement appeared homogeneous in healthy periodontium (Figure 4A), the damaged periodontal connective tissue showed an interlacing contrasting area, some displaying fibers parallel to the basal layer of the marginal epithelium; others, fiber-deprived, were rather toward the top of the gingival ridge (Figure 4B).

#### 3.4.3 Spectral Analysis of the Deep Periodontium (Cementum, PDL, Alveolar Bone)

The root-to-alveolar bone joint was thoroughly investigated using our approaches. A thin, regular cementum, typical of a healthy, deep periodontium (Figure 4A), can be distinguished from the underlying dentin. In this context, numerous osteocytic lacunae and intraosseous vascular channels are clearly visible in non-resorbed and corticalized alveolar bone. PDL fibers aligned orthogonally to the root surface, with their Sharpey fibers embedded in the cementum and alveolar bone, can be easily pointed out. The different types of deep periodontal lesions can also be identified. Images of large, fibrosis PDL displaying disorganized fibers between bone and cementum, often resorbed and typical of infra-bony lesions, may be reported. In other sites, areas of cementum-alveolar ankyloses, typical of diseased canine periodontium, were clearly recognized. Finally, spectral analysis seems suitable for the description of pathological supra-crestal attachments at the bottom of periodontal pockets, displaying typical long junctional epithelium, heterogeneous underlying connective tissue, and enlarged PDL, often associated with resorbed bone and cementum surfaces (Figure 4B).

## 4 Discussion

The PCA-based framework for composite hard/soft tissue spectral fluorescence image acquisitions described here is fast, modular, inexpensive and easy to implement. It provides an excellent cost-effectiveness ratio, with an obvious benefit to the user to investigate periodontal tissues and, by extension, complex, whole, intricate hard/soft tissues. This technical approach is not static: every stage of the protocol (fluorescence staining, preprocessing, and dimensionality reduction step) can be customized according to tissue types or the pathophysiological questions to be addressed. Moreover, conventional histology thin demineralized slices-based may follow tissue spectral fluorescence image acquisitions if needed.

We did not include any labeling agent in the UV channel, shown to be the only one to *per se* highlight mineralized tissues. Thus, the signal is related to both natural autofluorescence and the marginal contribution of crossover dyes (long-pass filters, Supplementary Table S2). A differential autofluorescence emission signal between a young and an older bone has been demonstrated after an excitation-light wavelength of around 350 nm (Prentice, 1967). This phenomenon may be attributed to the cellular components of the different tissue compartments, as well as to the collagen content (Fauch et al., 2019).

Since immunostaining is not possible on such a sample, use of fluorescent probes was mandatory. However, the use of fluorescent dyes on undecalcified sections, especially in large specimens requiring specific histological treatment such as long-time fixation and reslicing, is not commonly reported (Cannon et al., 1982). The mix of fluorescent dyes used here was set up for the particular case of the periodontal tissues. CFDA-SE, Calcein-AM, WGA, SRB, and TO-PRO3 are commonly used to label proteins and cell nuclei in tissue sections (Haugland, 1992; Bink et al., 2001; Kostrominova, 2011). In the present protocol, they provide green and red fluorescence mainly distributed in canine periodontal soft tissue. Obviously, the final staining mix cannot be defined as an algebraic contribution of each dye but as a unique combination capable by differential contrast enhancement reinforced by an algorithmic processing, to make a thorough histopathological analysis. Such a technique can therefore be adapted to different types of dyes and their mixture must be optimized according to the study sample.

The advent of widely available machine learning has brought a powerful set of tools for image processing. Among them, PCA, which is based on the reduction of dimensionality by a linear transformation (Franchi and Angulo, 2016; Ma et al., 2021), makes it possible to identify spectral signatures or features in the image in a fast and simple way to implement (Franchi and Angulo, 2016; Ma et al., 2021). Moreover, this approach could be extended to 3D acquisitions and generalized by increasing the number of spectral acquisitions (according to both excitation and emission wavelengths) and by integrating spatial–spectral feature extraction methods to consider both spectral and contextual information (Franchi and Angulo, 2016). Our objective in this work was to show the feasibility of the approach using simple, inexpensive, and fast tools and a fast process while remaining scalable.

Optical microscopy is particularly optimized whatever the magnification, with benefits for enhancing structures weakly highlighted in autofluorescence. Translated to higher magnification with high-resolution microscopes could even accurately discriminate cell subpopulations, especially combined with specific markers, such as directed to leukocytes or endothelium. Moreover, an easier and faster sample preparation compared to decalcification for histology and clearing for LSFM are obvious. For the same magnification, it is also faster than X-ray microscopy (from 5 to 30 h of acquisition length) while allowing a convenient analysis of both hard and soft tissues. Using this manipulation, the information is not lost at the mineral and anatomical levels compared to decalcified histology. Therefore, other microscopic approaches can be used downstream, and this approach can be integrated into an imaging pipeline.

Overall, this study enriches the PCA protocols previously developed for microscopy (Qu et al., 2002), spectral and hyperspectral, for *in vitro* cells and *in vivo* soft tissue investigations (Kong et al., 2004; Huang et al., 2005; Jo et al., 2006; Jayanthi et al., 2011). However, this strategy has never been optimized to improve discrimination of different cells/tissues phenotypes juxtaposed (and even mixed) inside a same sample, especially in large specimens. Here, we aimed to develop an imaging framework as a convenient strategy for optical microscopy investigation of large sample, including composite soft/mineralized tissue. We demonstrate here that eigen-images derived from both Std and Clahe pre-preprocessing significantly enhance contrasts for histopathological investigation with an *in-depth* detail for sub-tissular structure discrimination. Beyond periodontal investigations, this first proof of concept of an sPCA solution for optical microscopy will open new perspectives to deal with other chronic diseases involving complex tissues and organ defects.

## Data Availability

The code presented in this study and an example dataset can be found in online repositories : https://github.com/paulmonsarrat/spectral_periodontal_tissues.
