# Supplementary material for: A Spectral Principal Component Analysis-Based Framework for Composite Hard/Soft Tissue Fluorescence Image Investigation
Source: Front Physiol. 2022 Jul 13;13:899626. doi: 10.3389/fphys.2022.899626 (PMC9325997; doi:10.3389/fphys.2022.899626)
Supplement: Supplementary file 1 [file Presentation1.pdf]

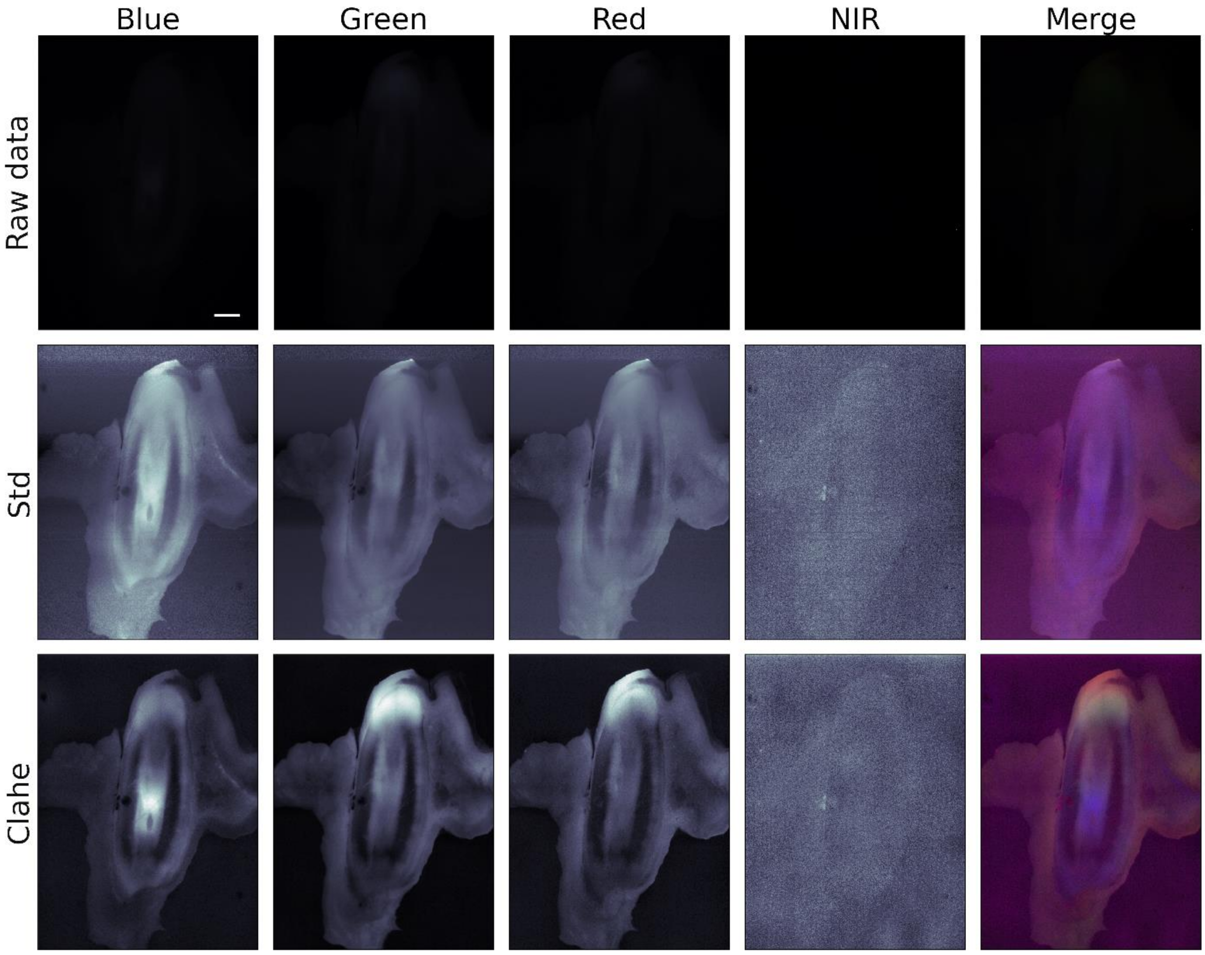

Fig. S1

Autofluorescence on the blue, green, red, and NIR channels before (raw) and after preprocessing (Std and Clahe). Scale bar : 2000 µm.

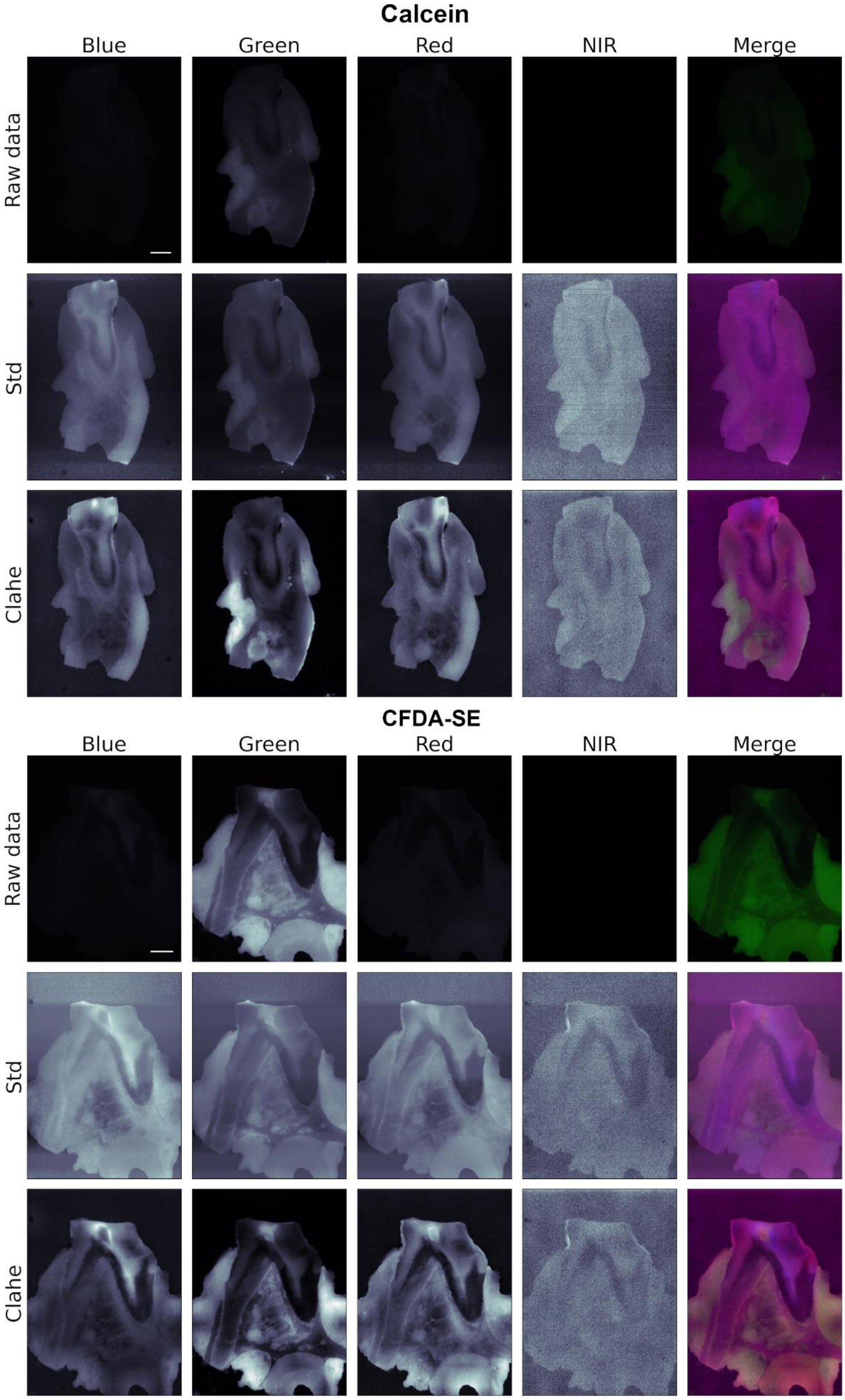

Fig. S2

Single-dye labeling (Calcein and CFDA-SE) on the blue, green, red. and NIR channels before (raw) and after preprocessing (Std and Clahe). Scale bar : 2000 µm.

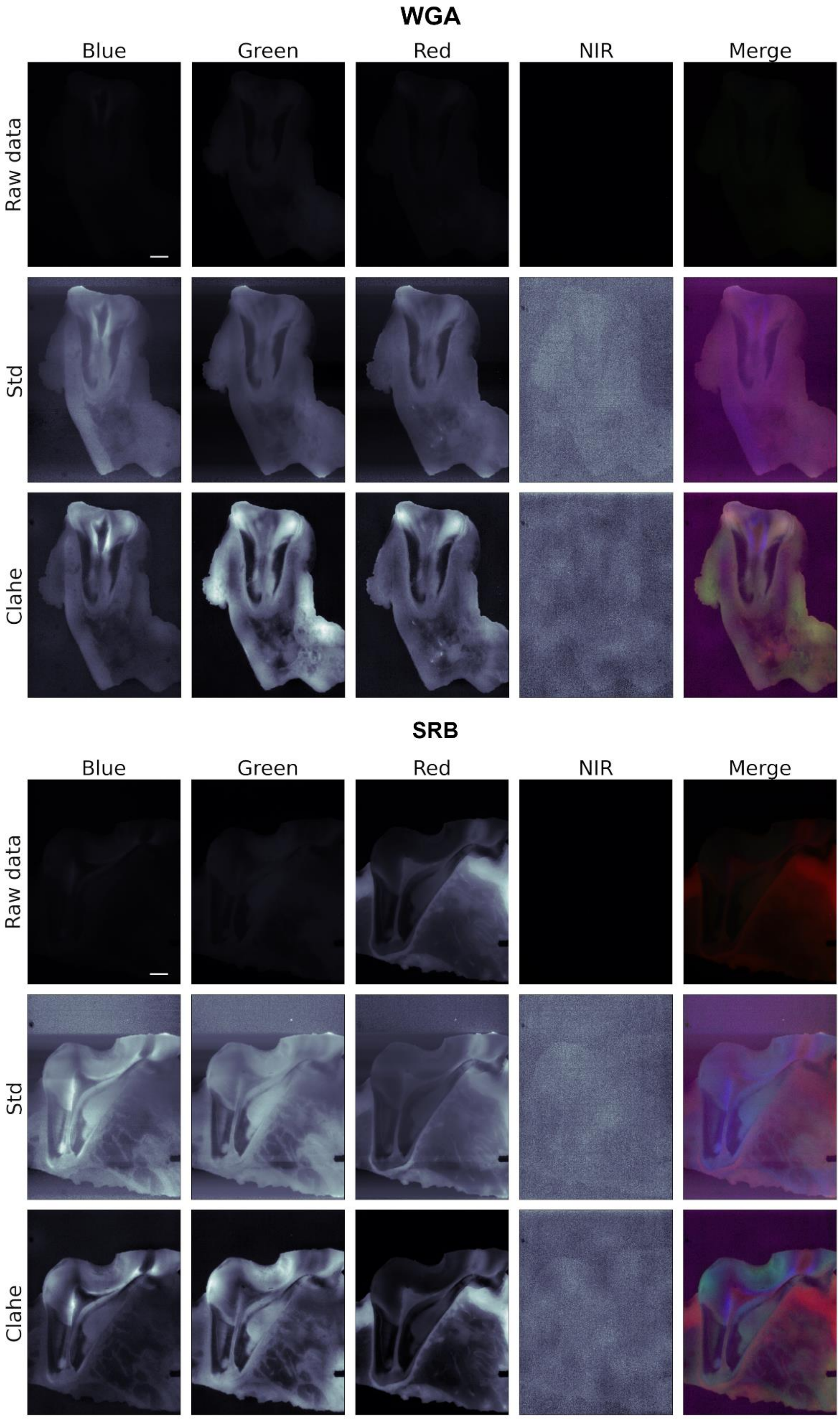
WGA
Blue
Green
Red
NIR
Merge
Raw data
Std
Clahe
SRB
Blue
Green
Red
NIR
Merge
Raw data
Std
Clahe

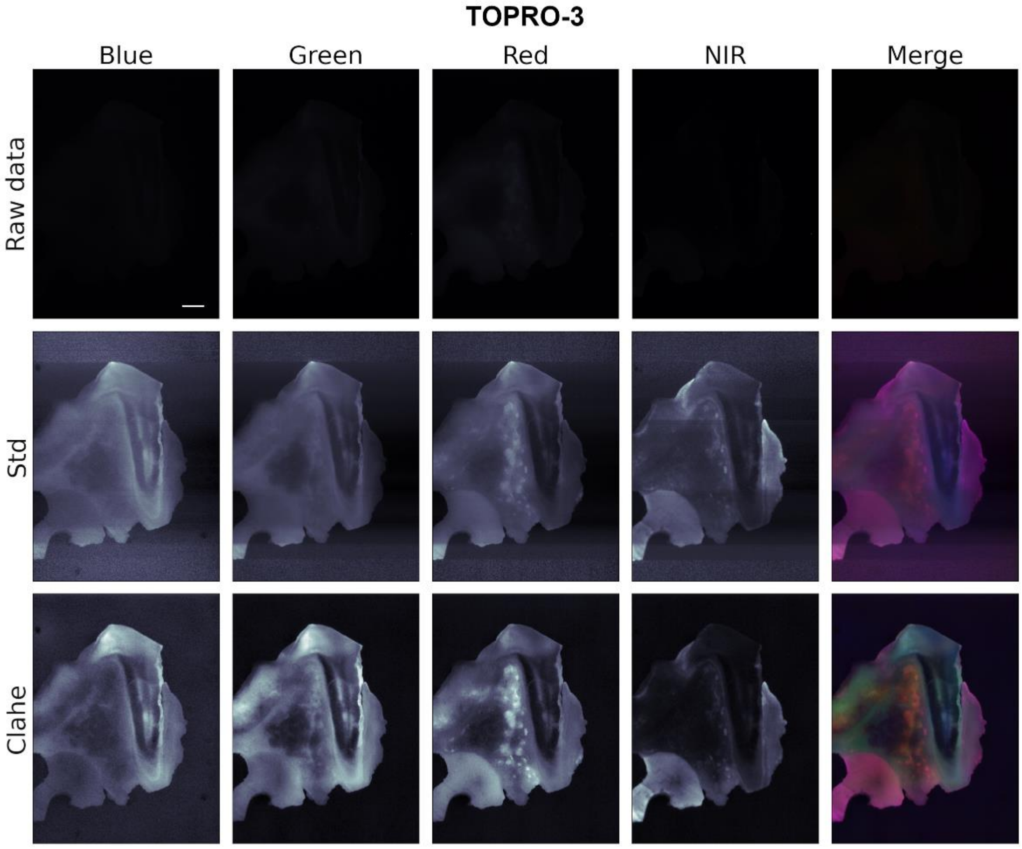

Fig. S3

Single-dye labeling (WGA, SRB and TOPRO-3) on the blue, green, red, and NIR channels before (raw) and after preprocessing (Std and Clahe). Scale bar : 2000 µm.

Table S1

Dye characteristics

Dye	Reference	Wavelength λ_{ex} / λ_{em} (nm)	Dilution
Sulforhodamine B	S1402-16 (Sigma Aldrich)	565 / 586	2 µg/ml
Calcein-AM	65-0853-78 ThermoFisher Scientific)	495 / 515	50 µM
TO-PRO™-3 Iodide	T3605 (ThermoFisher Scientific)	642 / 661	2 µM
Wheat Germ Agglutinin-Texas Red™ - X conjugate	W21405 (ThermoFisher Scientific)	595 / 615	2 µg/ml
5(6)-CFDA, SE; CFSE (5-(and-6)-Carboxyfluorescein Diacetate, Succinimidyl Ester), mixed isomers	C1157 (ThermoFisher Scientific)	492 / 517	200 µM

Table S2

Acquisition settings

	Cube Filter		Time exposure (ms)
	Excitation (nm)	Emission (nm)	
Blue	350/50	LP 420	1500
Green	470/40	525/50	1000
Red	540/25	605/55	500
Near Infrared	665/45	725/50	2000
